# Bad Things Come to Those Who Do Not Wait: Temporal Discounting Is Associated With Compulsive Overeating, Eating Disorder Psychopathology and Food Addiction

**DOI:** 10.3389/fpsyt.2019.00978

**Published:** 2020-01-22

**Authors:** Maria Kekic, Jessica McClelland, Savani Bartholdy, Rifka Chamali, Iain C. Campbell, Ulrike Schmidt

**Affiliations:** Institute of Psychiatry, Psychology & Neuroscience, King's College London, London, United Kingdom

**Keywords:** compulsive overeating, food addiction, obesity, temporal discounting, impulsivity

## Abstract

The tendency to act on immediate pleasure-driven desires, due to the devaluation of future rewards [a process known as temporal discounting (TD)], has been associated with substance use disorders (SUD) and with conditions characterised by compulsive overeating. The study involved a large inclusive participant sample (i.e., no diagnostic or exclusion criteria were applied). They were recruited/assessed online and we investigated whether TD was related to compulsive overeating and associated problems. Participants [*N* = 432, (48 males)] completed an online survey, which included a hypothetical monetary TD task, the Eating Disorder Examination-Questionnaire (EDE-Q), the Yale Food Addiction Scale (YFAS) and the Depression Anxiety and Stress Scales (DASS). TD correlated with frequency of compulsive overeating and compensatory behaviours, with eating disorder psychopathology, with scores on the YFAS, and with body mass index (BMI). As our study shows that elevated rates of TD are associated with a range of behaviours/measures, we propose that it is more likely that elevated TD rates are a predisposing factor rather than a consequence of the behaviour, i.e., elevated rates of TD contribute to pathological eating-related behaviours; however, a bi-directional explanation is also possible. Future research should investigate whether interventions aimed at reducing TD have clinical potential for treating problematic eating behaviours.

## Introduction

Compulsive overeating, binge-eating, and loss-of-control eating are behaviours used to describe the aberrant patterns of feeding that characterise the binge-purge subtype of anorexia nervosa (AN), bulimia nervosa (BN), binge-eating disorder (BED) and some forms of obesity Thus, compulsive overeating is not a diagnosis, rather, it is an umbrella term in common parlance. It refers to excessive food consumption that is accompanied by a perceived loss of control (LOC) over intake—not necessarily in a discrete period of time, as per binge-eating disorder (BED) (1). Compulsive overeating is sometimes accompanied by unhealthy compensatory behaviours such as self-induced vomiting and laxative misuse. Compulsive overeating, like other compulsive behaviours, can be broadly defined as a trait in which actions are persistently repeated despite adverse consequences (2). As such, it can have serious health consequences: conditions encompassing this behaviour are associated with medical problems (e.g., obesity and diabetes), psychiatric morbidity (e.g., emotional distress and affective illness), and with significant functional impairment (e.g., mobility) (3–5). Our participants did not have to have any eating disorder (ED) diagnosis and in our study, the term compulsive overeating was used to describe a category of behaviour.

The cause(s) of compulsive overeating are unclear. It has been proposed that in some individuals, compulsive overeating may result from a physical and psychological dependence on certain foods, i.e., highly palatable, energy-dense foods may have addictive properties similar to drugs of abuse (6). Indeed, parameters used to describe compulsive overeating mirror the Diagnostic and Statistical Manual of Mental Disorders (DSM), Fifth Edition criteria for substance use disorder (SUD) (1). Thus, people who compulsively overeat report experiencing intense food cravings; food is often consumed in larger amounts than intended; unsuccessful efforts to cut down or control one's overeating are commonplace; lastly, dysregulated eating continues despite knowledge of it being a physical or psychological problem that is likely to have been caused by this behaviour. Behavioural/neurobiological and neurocognitive studies that provide some support for “food addiction” are described below; however, although there are overlaps in the clinical characteristics of food and substance addiction (7, 8), whether food can actually be addictive remains a subject of debate (9).

There are overlaps in brain regions involved in responding to food and drug cues, e.g., in areas that are involved in reward processing including the striatum, amygdala, and anterior insula (10, for rev) and secondly, for the involvement of dopaminergic systems in these areas in relation to susceptibility to diet induced obesity (11). Furthermore, compulsive overeating and drug abuse are associated with reward-circuitry dysfunction, e.g., reward system hyper-responsivity during exposure to high-calorie tastes has been associated with compulsive overeating behaviour in overweight and obese individuals (12), which is similar to what has been reported in response to the presence of drug-related cues in SUD (13, 14). Results are also broadly consistent with the proposal that repeated exposure to potentially addictive substances renders reward circuits hypersensitive to associated stimuli or cues (15, 16).

Neurocognitive similarities also exist between people who are compulsive overeaters and substance abusers. Both groups appear to have deficits in temporal discounting (TD)—an aspect of impulse control defined as “the observed tendency for the value of reinforcers to decrease as a function of the delay to their delivery” (17). The concept of TD originated from the well known Stanford Marshmallow Test in which young children could choose to eat one marshmallow immediately or wait indefinitely for two marshmallows (18). Contemporary TD tasks typically consist of multiple binary choices between smaller rewards available sooner and larger rewards available later. A stronger preference for smaller-sooner (SS) rewards denotes a greater degree of TD, which is considered to reflect higher impulsivity and lower self-control.

People with BN, BED and obesity, [like those with SUD (19, 20)] are reported to exhibit steeper rates of TD compared to healthy controls (21–29). In drug addiction, TD has also been used to gauge illness severity and treatment outcomes (19, 20). In relation to problematic eating behaviour, an increased propensity to act on immediate pleasure-driven desires has been linked to greater energy intake (30), to a higher probability of binge-eating (30), and poorer treatment response (31) in people who are overweight/obese, to increased eating disorder (ED) psychopathology in heavy drinkers (32), and to a higher BMI in the general population (32–35). Lastly, in people who frequently crave food, the tendency to discount the value of delayed rewards is reported to negatively influence susceptibility to the anti-craving effects of neuromodulation (36).

Research into neurocognitive markers of disordered eating has usually involved participants being separated/investigated as diagnostic groups. However, problems in executive function (including impulse control) may be better investigated across a spectrum of pathological eating behaviour, as they appear to be present transdiagnostically and also in subclinical cases (37, 38). Accordingly, we examined whether TD was linearly related to compulsive overeating and associated psychological and physiological disturbances. We recruited a large inclusive online sample and took into account a number of potential confounding variables. We hypothesised that increased rates of TD would be associated with a) increases in the frequency of compulsive overeating and compensatory behaviours, b) the severity of ED psychopathology, c) “food addiction”, and d) BMI. We predicted these relationships would be independent of differences in demographic (age, sex, income, and education) and clinical (depression, anxiety, and stress) factors reported to influence TD behaviour (39–45).

## Materials and Methods

### Participants

Male and female volunteers (≥18 years of age) were invited to complete an online survey investigating “impulsivity in ED and obesity” for the chance to win an Apple^®^ iPad Mini™. They were recruited *via* online advertisements on a number of research recruitment webpages—including those of King's College London (KCL), Beat™ (Eating Disorders Association, United Kingdom), and the In-Mind Foundation—as well as on social media (Twitter^®^ and Facebook^®^). Importantly, the advertisements stated that individuals could participate regardless of whether they had been diagnosed with an ED, i.e., the study involved a large inclusive participant sample, i.e., no diagnostic or exclusion criteria were applied. They were recruited/assessed online and we investigated whether TD was related to compulsive overeating and associated problems. Questionaires were completed before the TD task. A total of 870 surveys were started and 432 (49.7%) of these were completed (perhaps due to the survey's relatively lengthy nature; data from uncompleted surveys are available on request).

This study was approved by the KCL Nursing and Midwifery Research Ethics Subcommittee, and data were obtained in compliance with KCL regulations. All participants were required to give electronic informed consent before they could gain access to the survey.

### Measures

#### Demographics Questionnaire

This 23-42-item (19 items were response-dependent) self-report survey of general descriptive information included questions relating to sex, age, height, weight, ethnicity, income, education, and ED status. BMI = weight (kg)/(height (m)^2^. was calculated for each participant.

#### Eating Disorder Examination-Questionnaire (EDE-Q) Version 6.0

This 33-item self-report inventory, derived from the EDE investigator-based interview, measures specific ED psychopathology over the past 28 days (46). It generates frequency data on key behavioural features of ED (e.g., compulsive overeating, vomiting, laxative misuse, over-exercising) and four subscale scores reflecting the severity of aspects of ED psychopathology (restraint, eating concern, shape concern, and weight concern). A global score is obtained by averaging subscale scores. Responses to items addressing ED severity are made on a 7-point scale (0–6), with scores ≥ 4 surpassing the recommended clinically relevant cut-off points (47). EDE-Q frequency data were used (in addition to subscale and global scores) to quantify compulsive overeating episodes and compensatory behaviours. The frequency of compulsive overeating episodes was defined as the number of times participants reported eating “what other people would regard as an unusually large amount of food, given the circumstances” accompanied by a “sense of having lost control over (their) eating, at the time (they) were eating” during the past 28 days (item 14). Frequency of compensatory behaviours was defined as the number of times participants reported making themselves “sick (vomit)…/taken laxatives as a means of controlling (their) shape or weight” or “exercised in a driven or compulsive way as a means of controlling (their) weight, shape or amount of fat, or to burn off calories” during the past 28 days (sum of items 16, 17, and 18).

#### Yale Food Addiction Scale (YFAS)

This self-report questionnaire measures addictive eating behaviour within the past 12 months (48). It contains 25 items (17 five-point frequency scales and eight binary choices) which map on to the DSM, 4th Edition, Text Revision criteria for substance dependence. It provides a continuous symptom count (0–7) and a dichotomous score (yes or no) denoting whether its criteria for food addiction have been met (a “diagnosis” is given if at least three symptoms and a clinically significant impairment or distress are present).

#### Depression Anxiety and Stress Scales-21 (DASS-21)

This self-report inventory assesses the frequency/severity of negative emotions experienced during the previous week (49). It consists of three 7-item subscales that measure depression, anxiety, and stress. Responses are made on a 4-point scale (0–3), and a separate score is generated for each dimension by summing values for relevant items and multiplying the total by 2. Scores indicate moderate to extremely severe levels of depression, anxiety and stress.

#### Temporal Discounting (TD) Task

TD behaviour was assessed with a hypothetical monetary choice task modelled on a paradigm developed previously (50, 51). Eighty binary choices were administered in succession: for each one, participants indicated whether they would prefer to receive a smaller amount of money available immediately (SS reward) or a larger amount available after a 3-month time delay [larger-later (LL) reward]. Two types of decision framing were employed: Accelerate and Delay. In the Accelerate set, the LL reward remained at £100 and the SS reward increased from £20 to £98 in £2 increments, resulting in a total of 40 binary choices. Alternatively, in the Delay set, the SS reward was fixed at £50 while the LL reward increased from £52 to £130 in £2 increments, also resulting in a total of 40 binary choices. The 80 choices were presented in a random order, which was the same for each participant.

TD was quantified by determining participants' discount factor (DF)—the magnitude of reduction in the present value of a future reward—for each choice set using a two-step procedure (23, 36, 50) (global DF was also calculated as the mean of the two DFs). First, the “indifference point” was established. This is the amount of money that the participant judged as equivalent to the fixed reward—i.e., the value of the variable reward when the participant switched from LL to SS in the Accelerate set and from SS to LL in the Delay set (50). Second, a formula was fitted to the indifference point: *δ* = (*x*_1_/*x*_2_)^(1/(^ *^t^* ^2−^ *^t^* ^1))^, where *x*_1_ is the SS reward, *x*_2_ is the LL reward, and *t*_2_-*t*_1_ is the delay to reward presentation (in years), which in this case was 0.25 (50, 51) This is a sensitive measure of TD that is independent of hyperbolic modelling and area under the curve analyses (50, 51)The values obtained can range from 0 to 1, with smaller numbers indicating greater TD and thus a greater tendency to choose the immediate reward. When no indifference point was calculable (i.e., when no switch was made between SS and LL rewards) a default score was assigned: 0 if the SS reward was always selected and 1 if the LL reward was always chosen.

### Data Analysis

Statistical analyses were performed, according to recommendations from a biostatistician at KCL, using IBM^®^ SPSS^®^ software (Version 22). All tests were two-tailed: the level of significance was set at α = 0.05.

Inspection of histograms indicated that TD data were positively skewed. Square-root transformations were performed to Accelerate and Delay DFs, and these were effective in normalising the data. The global DF was then calculated as the mean of the transformed scores (GlobalSqrtDF). To test for potential confounders, a series of multiple linear regressions were conducted with GlobalSqrtDF as the dependent variable. Age, annual personal income, highest level of education, sex, and depression, anxiety, and stress DASS-21 subscale scores were entered as independent variables (the former three were dummy coded) in a forward manner based on statistical significance. Since no model was able to predict changes in GlobalSqrtDF (*R* ^2^ < 0.06, *p* > 0.10 for all models), these variables were excluded from further analyses.

Bivariate correlations were used to investigate the relationships between raw DFs, the frequency of compulsive overeating episodes and compensatory behaviours (as reported in the EDE-Q), subscale and global EDE-Q scores, continuous YFAS score, and BMI. Pearson's r (parametric) and Spearman's rho (non-parametric) correlation coefficients were employed.

## Results

### Demographic Characteristics

For the income and education variables, “prefer not to say” (*n* = 77) and “other” (*n* = 56) responses were coded as missing values, respectively. When there were obvious reporting errors, e.g., height values outside the expected range, these were removed from the dataset (*n* = 16). [These were defined as observations lying ±3 SD from the average adult male (177.2 cm, *SD* = 8.1) and female (163.0 cm, *SD* = 7.8) heights in the UK (52)]. To eliminate the remaining suspected erroneous BMI data, entries ±3 SD from the sample mean (*M* = 25.71, *SD* = 10.45) were discarded (*n* = 6). National averages were not used here as the sample contained individuals with extreme weight conditions (AN and BED).

The sample consisted of 384 females and 48 males: 52.8% were aged between 18–24 and 70.4% self-defined their ethnicity as “white”. After presumed erroneous entries were removed (*n* = 22, see above), the mean BMI [weight (kg)/(height (m)^2^] was 24.91 (*SD* = 7.76, range: 12.76-56.80): 12.2% of participants were underweight (<18.50); 55.4% were of normal weight (18.50–24.99); 13.2% were overweight (25–29.99); and 19.3% were obese (≥30) (NHS, 2014). Of the participants who declared their annual personal income (*n* = 355), 60.3% earned <£20,000 and, of those who specified their highest level of education (*n* = 376), 65.7% had Higher Education qualifications. More detailed demographic information is provided in **Table 1**.

**Table 1:** Demographic characteristics.

	*n* (M)	% (SD)
**Sex**	432	100
Male	48	11.1
Female	384	88.9
**Age**	432	100
18–24	228	52.8
25–34	138	31.9
35–44	36	8.3
45–54	20	4.6
55–64	6	1.4
65+	4	0.9
**Ethnicity**	432	100
White	304	70.4
Mixed	23	5.3
Asian	44	10.2
Black	20	4.6
Arab	29	6.7
Other	12	2.8
**Annual personal income**	355	82.2
<£20,000	214	60.3
£20,000–£39,000	94	26.5
£40,000–£59,999	19	5.4
£60,000–£99,999	15	4.2
>£100,000	13	3.7
**Highest level of education** ^a^	376	87.0
No qualifications	17	4.5
Secondary/Further Education	112	29.8
Higher Education	247	65.7
**BMI**	410 (24.91)	94.9 (7.76)
Underweight	50 (16.85)	12.2 (1.37)
Normal weight	227 (21.55)	55.4 (1.79)
Overweight	54 (27.16)	13.2 (1.49)
Obese	79 (38.14)	19.3 (6.96)
**Self-reported eating disorder diagnosis**	432	100
No eating disorder	301	69.7
Anorexia nervosa	42	9.7
Bulimia nervosa	36	8.3
Binge eating disorder	24	5.6
Other	29	6.7

M, mean; SD, standard deviation; BMI, body mass index.

a Categories used correspond to UK Education System.

### Clinical Features and Temporal Discounting

**Table 2** summarises scores on the TD task and outcomes for each of the clinical measures in the survey. The full range of possible scores was observed across all scales excepting the YFAS continuous symptom count, for which the maximum value of seven was not obtained by any participant. The mean global EDE-Q score was 2.94 (*SD* = 1.70), and 32.6% of participants had clinically relevant scores (≥4; Rø et al., 2012). One third of participants (33.1%) met criteria for food addiction according to the YFAS [see above; (48), and the mean continuous score for the sample as a whole was 2.88 (*SD* = 1.87)]. Mean DASS-21 scores were within the moderate range for depression (14–20; *M* = 16.25, *SD* = 13.42) and anxiety (10–14; *M* = 12.25, *SD* = 11.00), and within the mild range for stress (15–18; *M* = 16.25, *SD* = 13.42). Severe or extremely severe levels of depression (≥21), anxiety (≥15), and stress (≥26) were reported by 38.2%, 37.3%, and 31.0% of participants, respectively. The mean DF was 0.37 for the Accelerate set and 0.34 for the Delay set. The difference between these scores was significant (*z* = -4.72, *p* < .001): individuals discounted the future reward more when asked to delay consumption than when given the opportunity to accelerate consumption.

**Table 2:** Clinical features and temporal discounting.

	Mean	*SD*	Range
**EDE-Q**	–	–	–
Restraint	2.61	1.80	0.00–6.00
Eating concern	2.25	1.82	0.00–6.00
Shape concern	3.65	1.86	0.00–6.00
Weight concern	3.26	1.89	0.00–6.00
Global	2.94	1.70	0.00–6.00
Number of compulsive overeating episodes^a^	6.13	12.88	0.00–100.00
Number of compensatory behaviours^b^	11.44	24.20	0.00–295.00
**YFAS**	–	–	–
Continuous	2.88	1.87	0.00–6.00
Dichotomous (yes/no)	*n* = 143/289	% = 33.1/66.9	–
**DASS-21**	–	–	–
Depression	16.25	13.42	0.00–42.00
Anxiety	12.25	11.00	0.00–42.00
Stress	17.13	12.32	0.00–42.00
**Temporal discounting (DF)^c^**	–	–	–
Accelerate	0.37	0.33	0.00–1.00
Delay	0.34	0.29	0.00–1.00
Global	0.35	0.29	0.00–1.00
Sqrt accelerate	0.52	0.30	0.00–1.00
Sqrt delay	0.52	0.26	0.00–1.00
Sqrt global	0.52	0.26	0.00–1.00

SD, standard deviation; EDE-Q, Eating Disorders Examination-Questionnaire; YFAS, Yale Food Addiction Scale; DASS-21, Depression Anxiety and Stress Scales 21; DF, discount factor; Sqrt, square-root transformed.

a EDE-Q item 14: “On how many of these times did you have a sense of having lost control over your eating, at the time you were eating?” (Follows the question “Over the past 28 days, how many times have you eaten what other people would regard as an unusually large amount of food, given the circumstances)?

b Sum of EDE-Q items 16, 17 and 18: “Over the past 28 days, how many times have you [Item 16] made yourself sick (vomit) as a means of controlling your shape or weight/[Item 17] taken laxatives as a means of controlling your shape or weight/[Item 18] exercised in a driven or compulsive way as a means of controlling your weight, shape or amount of fat, or to burn off calories?

c Smaller discount factors indicate greater temporal discounting.

Correlations between TD rates and other variables are shown in **Table 3**. Accelerate DF and Delay DF were highly positively and significantly correlated. Furthermore, all eating-related outcomes (EDE-Q scores, EDE-Q frequency data, the YFAS continuous score) were significantly positively interrelated with each other. Global DF was modestly but significantly negatively related to the frequency of compulsive overeating episodes and with compensatory behaviours (as reported in the EDE-Q). Global DF was also modestly but significantly negatively related, to eating, shape and weight concern (EDE-Q subscale scores), to the global EDE-Q score, to the YFAS symptom count, and to BMI. Lastly, a greater tendency to choose the SS reward (increased TD) was associated with higher scores across these outcome measures.

**Table 3:** Correlations between temporal discounting rates and clinical variables.

		1	2	3	4	5	6	7	8	9	10	11
**1**	Accelerate DF^a^	–	–	–	–	–	–	–	–	–	–	–
**2**	Delay DF^a^	.71**	–	–	–	–	–	–	–	–	–	–
**3**	Global DF^a^	.90**	.94**	–	–	–	–	–	–	–	–	–
**4**	EDE-Q restraint	-.04	-.08	-.08	–	–	–	–	–	–	–	–
**5**	EDE-Q eating concern	-.15**	-.16**	-.17**	.73**	–	–	–	–	–	–	–
**6**	EDE-Q shape concern	-.14**	-.18**	-.18**	.75**	.82**	–	–	–	–	–	–
**7**	EDE-Q weight concern	-.14**	-.16**	-.18**	.73**	.84**	.94**	–	–	–	–	–
**8**	EDE-Q global	-.12*	-.15**	-.16**	.87**	.92**	.95**	.95**	–	–	–	–
**9**	Compulsive overeating episodes^b^	-.11*	-.11*	-.12*	.39**	.59**	.50**	.50**	.53**	–	–	–
**10**	Compensatory behaviours	-.12*	-.11*	-.12*	.60**	.54**	.53**	.49**	.58**	.32**	–	–
**11**	YFAS continuous	-.17**	-.13**	-.16**	.45**	.70**	.62**	.62**	.65**	.57**	.35**	–
**12**	BMI^c^	-.09	-.10	-.10*	-.12*	-.05	-.00	.04	-.03	.13**	-.25**	.05

DF, discount factor; EDE-Q, Eating Disorder Examination-Questionnaire; YFAS, Yale Food Addiction Scale; BMI, body mass index.

Coefficients in normal text are Spearman's Rho; coefficients in bold text are Pearson's R. **p < 0.05; ***p < 0.01

^a^Smaller discount factors indicate greater temporal discounting; ^b^ n = 428; ^c^ n = 410

## Discussion

This research sought to extend studies examining relationships between TD and eating-related pathological behaviours. We used a large inclusive sample of participants (i.e., no diagnostic or exclusion criteria were applied). They were recruited and assessed online. Using our dimensional and symptom based (rather than diagnostic) approach, we found that an increase in TD rate were linearly associated with a) an increase in the frequency of compulsive overeating and compensatory behaviours, b) the severity of “food addiction”, c) ED psychopathology (eating, shape and weight concerns), and d) BMI. These findings were not confounded by demographic (age, sex, income, and education) or clinical (depression, anxiety, and stress) factors reported to affect TD behaviour (39–44). The results on measures of are consistent with literature reporting that excessive TD is involved in addictive behaviour and related disturbances (53–55) and consistent with our hypotheses.

The idea that excessive TD operates as an antecedent to compulsive overeating seems plausible, e.g., an individual may choose to overeat having decided that immediate gratification outweighs any adverse effects on future health and well-being. Several studies are consistent with this proposal. In a longitudinal study spanning mid-adolescence to young adulthood (56), it was reported that baseline TD predicted adoption of smoking in later life. A second study (57) demonstrated that male social drinkers who displayed steeper rates of TD when they entered a bar showed greater increases in blood alcohol levels when they exited. Thirdly, it has been reported (58) that 4-year-olds who were less able to delay gratification had higher BMI when followed-up approximately 30 years later.

Several models have been proposed to explain how the initiation of compulsive overeating could be related to. failures in self-control. According to one study (59), a disposition to act rashly when distressed is likely to increase the risk of impulsive engagement in bulimic behaviours when it is coupled with a psychosocial learning history that emphasises the benefits of eating and of thinness. On the other hand, it has been proposed that impaired self-regulatory control (underpinned by dysregulation of frontostriatal circuitry) interacts with hunger to release eating behaviour from control systems, and this results in binge eating (60). Lastly, it has been hypothesised that compulsive overeating results from an imbalance between “top-down” (cognitive) and “bottom-up” (appetitive) neural systems. In this scenario, exposure to relevant cues or ingestion of hyperpalatable foods would heighten activity in subcortical reward regions such that prefrontal self-control mechanisms will be less able to regulate behaviour (61): negative affect and resource depletion might amplify this effect (61).

Although TD rate is seen as a relatively stable personality trait, there is evidence that it can be modified by behavioural, pharmacological, and neuromodulatory techniques (for revs, see 62, 63). Moreover, experimentally induced improvements in TD behaviour have been reported to occur alongside reductions in ED symptoms in people with AN and BN and with energy intake in overweight/obesity (64, 65). Given the linearity of the relationships seen in this study, manipulations promoting adaptive intertemporal decision-making could also serve as preventative measures if implemented in at-risk groups. One approach could include a focus on early, pre-syndromal stages of illness as this time period is reported to be associated with better outcomes in ED and obesity (66–68).

As this study is cross-sectional, it does not allow for causal inferences. Another possible limitation is the self-report nature of its demographic data. For example, only 50% of participants completed the survey and secondly, the accuracy of BMI values may be somewhat compromised by the tendency of people to overestimate height and underestimate weight: however, such unidirectional response biases have little impact on correlational analyses. The clinical data were also obtained *via* self-report methods: however, the instruments used (DASS-21, EDE-Q, and YFAS) are standardised measures with adequate criterion and/or construct validity (48, 69, 70).

In relation to the TD task, evidence indicates that discounting rates for real and hypothetical rewards do not differ significantly, and that results from experiments with hypothetical rewards apply to everyday life (71). However, our paradigm may have been limited by its restriction to choices between immediate rewards and rewards delayed by three months, i.e., a replication study might be better if it involved using titration procedures that collect data across multiple delay periods. In this context, it is also of note that differences in TD paradigms also hinder comparisons between different TD studies, such as those related to SUD versus those investigating compulsive overeating.

Although we had no exclusion criteria, the location of the adverts and the topic (“impulsivity in ED and obesity”) may have biased recruitment towards particular individuals (e.g., university students and/or people with an ED/obesity). It is therefore not surprising that there was quite a high percentage of participants who met criteria for food addiction and also for severe depression. Similarly, the chance to win an Apple^®^ iPad Mini™ may have had more motivational value for certain groups (72). Thus, our sample may have been somewhat unrepresentative in terms of age, sex, income, and education, and this could have contributed to these variables' lack of influence over TD. Furthermore, in the context of the participant cohort, it could be argued that only participants with greater executive control would persevere with completing the online survey: however, this possible effect is likely to be cancelled out as the participants did not have a diagnosis and were compared with each other. Lastly, it is perhaps surprising that BMI and measures of TD are not significantly correlated: it is possible; however, that only certain phenotypes (especially those associated with loss-of-control or addictive eating) would show increased delay counting.

In summary, our study advances understanding of the relationships between TD and eating-related pathologies. We have replicated previous studies demonstrating linear associations between TD rate and a) ED psychopathology (based on the data from the EDE-Q), and b) BMI. We have also shown, that TD is unambiguously and linearly related to compulsive overeating, to compensatory behaviours, and lastly to “food addiction” based on scores from the YFAS).

Future studies should determine whether TD predisposes individuals to compulsive overeating and associated psychological and physiological disturbances, and whether prevention and intervention programmes aimed at helping people lower the rate at which they discount the value of future health benefits can induce clinically meaningful behavioural change.

## Data Availability Statement

The datasets generated for this study are available on request to the corresponding author.

## Ethics Statement

This study was approved by the KCL Nursing and Midwifery Research Ethics Subcommittee, and data were obtained in compliance with KCL regulations. All participants were required to give electronic informed consent before they could gain access to the survey. The patients/participants provided their written informed consent to participate in this study.

## Author Contributions

MK, JM, SB, and RC were involved in conducting the study. MK, JM, and SB were involved in analyzing the data and writing the paper. IC and US were involved in writing the paper and supervising the project.

## Funding

This work was supported by the Institute of Psychiatry, Psychology and Neuroscience/Medical Research Council (M.K., Excellence Studentship) and the National Institute for Health Research (NIHR), the Mental Health Biomedical Research Centre (BRC) at South London and Maudsley (SLaM), the National Health Service (NHS) Foundation Trust, and King's College London (KCL) (JM, SB, IC, US). The views expressed are those of the authors and not necessarily those of the NHS, the NIHR or the Department of Health.

## Conflict of Interest

The authors declare that the research was conducted in the absence of any commercial or financial relationships that could be construed as a potential conflict of interest.
